# *Aralia continentalis kitagawa* Extract Attenuates the Fatigue Induced by Exhaustive Exercise through Inhibition of Oxidative Stress

**DOI:** 10.3390/antiox9050379

**Published:** 2020-05-04

**Authors:** Dong Kwon Yang, Sei-Jin Lee, Gareeballah Osman Adam, Shang-Jin Kim

**Affiliations:** 1 Department of Veterinary Pharmacology and Toxicology, College of Veterinary Medicine, Jeonbuk National University, Iksan 54596, Korea; dkyang0502@gmail.com (D.K.Y.); gorba000@gmail.com (G.O.A.); 2 Korea Basic Science Institute Jeonju Center, Jeonju 54896, Korea; lsj@kbsi.re.kr

**Keywords:** *Aralia continentalis kitagawa*, fatigue, exhaustive exercise, oxidative stress, metabolic acidosis

## Abstract

The present study aimed to evaluate the anti-fatigue effects of *Aralia continentalis kitagawa* (AC) extract during exhaustive exercise of rats by forced swimming. Rats were subjected to forced swimming until exhausted after pre-treatment with AC extract for 21 days. Exhaustion time significantly increased in rats treated with AC extract. AC treatment also preserved blood homeostasis during fatigue due to exhaustive exercise. For fatigue-related serum biomarkers, AC extract significantly fail to decrease glucose and triglyceride (TG), but ameliorated increased lactate levels compared with levels in control rats. Metabolic acidosis, a major cause of fatigue, was effectively attenuated by AC extract, according to metabolic acidosis-related blood parameters. AC extract suppressed muscle injury and attenuated gastrocnemius muscle apoptotic responses due to exhaustive exercise. To investigate the mechanisms behind the AC extract anti-fatigue effect, we evaluated its effect on oxidative stress-related fatigue. We showed that pro-oxidants were inhibited, while antioxidants were preserved by AC extract treatment. Therefore, the anti-fatigue effect of AC extract was mediated by suppression of oxidative stress. Overall, the study demonstrated that AC extract effectively attenuates fatigue from exhaustive exercise through oxidative stress inhibition. AC extract, as an antioxidant, could be utilized as a therapeutic or preventive strategy against exhaustive exercise fatigue.

## 1. Introduction

Fatigue induced by exhaustive exercise is defined as a feeling of extreme physical or mental tiredness, weakness, or exhaustion [1]. The incidence of fatigue is closely associated with inflammation and chronic pain and can worsen a patient’s quality of life, especially when the disease is severe, or mild but persistent long term [2]. In addition, fatigue is closely related to sophisticated metabolic conditions caused by various factors such as the subject’s age, duration of the activity, and severity of the exercise [3]. During exercise, glycogen from the liver and muscle is metabolized to glucose by the phosphocreatine system, and glucose is further metabolized to satisfy the higher energy demands. Consequently, lactic acid accumulates in the body, especially within the muscles [4,5]. Lactic acid accumulation impairs the contractility and reduces the innervation of muscles, ultimately leading to muscle fatigue [6]. In addition, exhaustive exercise causes an imbalance in the energy demand, resulting in the body becoming exhausted when it persists. Changes in blood hemostatic parameters including pH, ions, and gases, also contribute to fatigue [7]. Hence, physical fatigue causes reduced performance through the accumulation of metabolic products including lipid peroxides, lactate, and ions [8,9].

The relationship between exhaustive exercise and oxidative stress is well established [10]. In fact, exhaustive exercise may cause the over-production of reactive oxygen species (ROS), leading to oxidative stress, although mild exercise improves body function. The accumulation of ROS further damages cell membranes, is deleterious to skeletal muscle performance, and causes fatigue during exhaustive exercise [10]. Therefore, supplementation with exogenous antioxidants is a promising strategy to protect body functions against fatigue during exhaustive exercise [11]. 

In addition, many medicinal plants and their antioxidant ingredients such as polysaccharides, alkaloids, and polyphenols have been reported to reduce pain, inflammation, and fatigue after high-intensity exercise [12]. Indeed, these plants contribute to improving exercise endurance and delaying exercise-induced fatigue through the activation of endogenous antioxidants and removal of superoxide radicals when given to mice before exhaustive swimming [13].

*Aralia continentalis kitagawa* (AC), belonging to the Araliacea family, is a medicinal herbal plant distributed widely throughout northeast Asia including China and Korea [14,15,16]. AC is used in traditional Korean medicine to relieve pain and inflammation [17]. Previous studies have found that AC possesses many pharmacological properties including anti-oxidative [14], hypocholesterolemic [18], and anti-diabetic effects [19]. Furthermore, AC has been reported to contain a variety of bioactive compounds including continentalic acid, epi-continentalic acid, and kaurenoic acid, which exhibit anticancer [20] and anti-inflammatory [17] activities. Various saponins, which have potential preventive effects against diabetes and hepatic injury, have also been isolated from AC [21,22]. In addition, recent studies have revealed that AC roots contain chlorogenic acid [23,24], a known antioxidant [25]. AC roots have been traditionally used to mitigate the pain, rheumatism, and inflammation that are often associated with fatigue [26,27]. However, the effects of AC and the underlying mechanisms on fatigue induced by exhaustive exercise have not yet been investigated. Therefore, in this study, we explored whether an AC water extract reduced the fatigue induced by exhaustive swimming, and further elucidated the underlying mechanisms of the anti-fatigue effect, focusing on the prevention of oxidative stress.

## 2. Materials and Methods

### 2.1. Preparation of Aralia Extract

The AC roots (1 kg) were purchased from a Korean traditional market, Jeonju, Korea. The roots of AC water extract were used in this study. Briefly, the roots of AC were dried in an incubator at 60 °C and powdered in an electric blender. The AC roots were extracted with water for 72 h with occasional stirring at room temperature for 1 h. The extracts were filtered using filter paper, evaporated in a rotary vacuum evaporator, and concentrated. Then, they were lyophilized with hot air-drying for 72 h at 50 °C. The total amount of AC extracted was 85 g. The water extracts of AC were kept at 4 °C for further study.

### 2.2. Ethics Statement

All animal procedures in this study were approved by the Committee on the Care of Laboratory Animal Resources of Jeonbuk National University (CBNU2016-67) and were performed in accordance with the Guide for the Care and Use of Laboratory Animals published by the U.S. National Institutes of Health (Bethesda, MA, USA; NIH Publication no. 85–23, revised 1996). Fifty male Sprague-Dawley rats (220–250 g; Samtako Bio Korea Co. Ltd., Daejeon, Korea) were employed in this study.

### 2.3. Animal Study Design

The rats were maintained at 23 ± 2 °C with 50 ± 5% humidity and a 12-h light/dark cycle in cages and acclimated for at least one week before experiments. Animals were divided into five groups (*n* = 10 in each group), where the control group received no exhaustive exercise without AC treatment, exhaustive exercise with saline as a vehicle-treated group, and exhaustive exercise with 60, 120, and 180 mg/kg AC extract-treated groups. AC extract was daily administered by oral gavage, respectively. On the third week after AC extract treatment, all animals except the control group were subjected to the forced swimming until exhaustion. For euthanasia, the rats were anesthetized with CO_2_ inhalation to minimize suffering.

### 2.4. Forced Swimming Test

The swimming pool for forced swimming was made of a glass chamber of 90 cm length, 60 cm width, and 70 cm height filled with water up to 55 cm. At the chamber basement, a thermostatic heater controller was used to maintain the water temperature at 36 ± 1 °C. The rats were subjected to individual forced swimming until exhaustion, which was considered as failure to return to the water surface for breathing within at least seven seconds. 

### 2.5. Quantification of Chlorogenic Acid in Aralia continentalis kitagawa (AC)

Chlorogenic acid were measured on a Kromasil 100-5 C18 column (4.6 mm × 250 mm) using a HPLC (high-performance liquid chromatography) system (Thermo electron Co., Beverly, MA, USA). A gradient elution was carried out with solvent A (formic acid:water = 10:90, *v/v*) and solvent B (acetonitrile:methanol:formic acid:water = 22.5:22.5:1.5:48.5, *v/v*) for the analysis of chlorogenic acid. The flow rate was 1.0 mL/min. Absorption spectrum of chlorogenic acid was recorded from 518 nm with an inline PDA detector.

### 2.6. Analysis of Blood and Serum Parameters

After forced-swimming, blood samples from the caudal vena cava were collected in lithium heparin-containing tubes. Serum was collected by centrifugation of whole blood samples at 3000× *rpm* for 10 min and kept at −80 °C for further studies. A Nova Stat Profile® pHOx Ultra Analyzer system (Nova Biomedical, Waltham, MA, USA) was used to measure blood pH, HCO_3_^−^, partial pressure of oxygen (*p*O_2_), the partial pressure of carbon dioxide (*p*CO_2_), lactate, hematocrit (Hct), magnesium (Mg^2+^), calcium (Ca^2+^), potassium (K^+^), and sodium (Na^+^). A Hitachi 7020 auto-analyzer (Hitachi, Tokyo, Japan) was also used for analyses of glucose, triglyceride (TG), creatine kinase (CK), and uric acid (UA) serum levels.

### 2.7. Measurement of Oxidative Stress-Related Proteins

Serum lactic dehydrogenase (LDH) was measured by using a Hitachi 7020 auto-analyzer (Hitachi, Tokyo, Japan). Lipid peroxidation was determined by measuring serum malondialdehyde (MDA) level using the OXI-TEK TBARS assay kit (Enzo Life Sciences Inc., Farmingdale, NY, USA), according to the manufacturer’s protocol. The serum levels of SOD and GSH were measured by using each SOD and GSH activity detection kit (Sigma-Aldrich, St. Louis, MO, USA), according to the manufacturer’s instruction.

### 2.8. Western Blot Analysis

Protein were prepared from gastrocnemius muscles using RIPA buffer containing inhibitor cocktail (Roche, Indianapolis, IN, USA) and phosphatase inhibitor cocktail (ThermoFisher Scientific Inc., Waltham, MA, USA). Protein samples were separated on SDS-PAGE and transferred to PVDF membranes (EMP Milipore Inc., Billerica, MA, USA), followed by blocking with 5% bovine serum albumin (Sigma, St. Louis, MO, USA) in TBST buffer at room temperature. The membranes were then incubated overnight at 4 °C with antibodies against Bax, Bcl-2, and pro-caspase 3, cleaved-caspase 3, and β-actin (Cell Signaling Tech., Danvers, MA, USA). The membranes were then incubated with the appropriate horseradish peroxidase-conjugated secondary antibodies (Cell Signaling Tech.) at room temperature for 1 h, followed by detection of signals using an Immobilon Western Chemiluminescence kit (Millipore Corp., Billerica, MA, USA) and a UVITEC Mini HD9 system (Cleaver Scientific Ltd., Warwickshire, UK). The intensity of each protein band was quantified using NIH ImageJ software (National Institute of Health, Bethesda, MD, USA).

### 2.9. Statistical Analysis

Statistical significance was analyzed using one-way analysis of variance (ANOVA) or Student’s t-test with Bonferroni post-hoc analysis for multiple group comparisons using GraphPad Prism 5.03 software (GraphPad Software Inc., San Diego, CA, USA). All data are reported as mean ± standard error of the mean (SEM). *P* < 0.05 was considered statistically significant.

## 3. Results

### 3.1. AC Extract Contains Chlorogenic Acid as an Active Component according to HPLC Analysis

**Figure 1:**
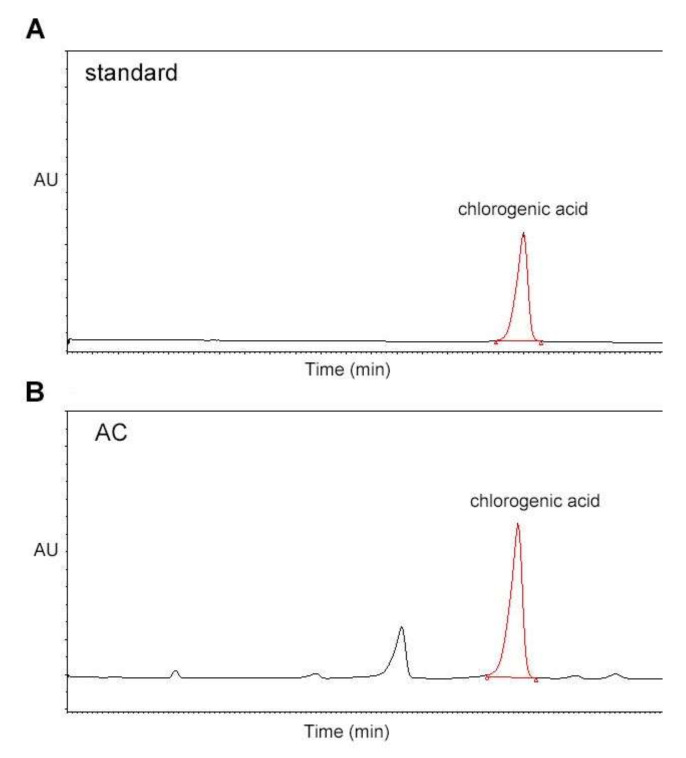
HPLC analysis of chlorogenic acid in the AC extract. HPLC was performed to determine the composition of chlorogenic acid in AC. The contents of chlorogenic acid were analyzed in (**A**) standard solution and (**B**) AC water extract. HPLC, high-performance liquid chromatography; AC, *Aralia continentalis kitagawa* extract.

Since previous studies have shown that AC extract contains chlorogenic acid as an active component [23,24], we sought to determine the composition of chlorogenic acid in the AC water extract using HPLC analysis. The presence of chlorogenic acid was confirmed by comparing its UV spectrum and retention time with that of a standard compound. The results showed that the AC water extract contained 2.24 mg/g chlorogenic acid (Figure 1).

### 3.2. AC Extract Increases Exercise Duration during Exhaustive Swimming

**Figure 2:**
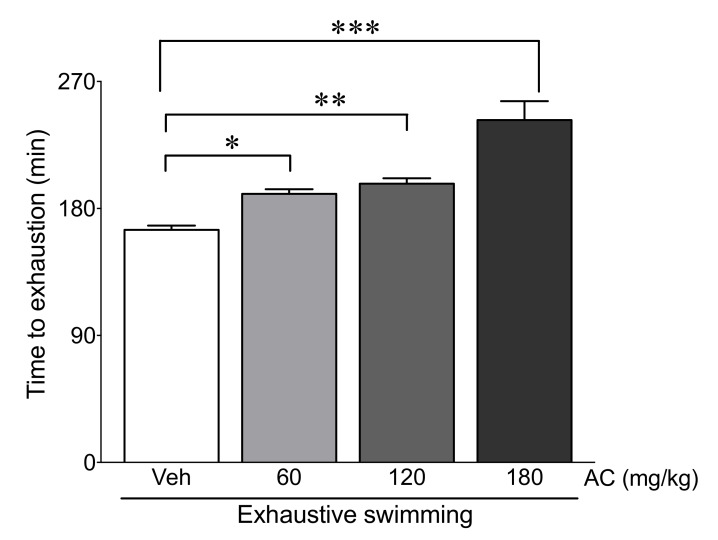
AC extract treatment enhanced the exercise duration of rats during exhaustive swimming. Exhaustion time was measured in rats after AC-pre-treatment for three weeks (*n* = 10 per group). Data are mean ± standard error of mean (SEM). Significance was measured using one-way analysis of variance (ANOVA) followed by Bonferroni’s post hoc test. * *p* < 0.05, ** *p* < 0.01, and *** *p* < 0.001. Veh, vehicle-treated; 60, 120, and 180 AC, 60, 120, 180 mg/kg AC extract-treated groups, respectively. AC, *Aralia continentalis kitagawa* extract.

To examine the effects of the AC extract on exhaustive exercise duration, AC- and vehicle-treated rats were subjected to the forced-swimming test in the swimming pool. As shown in Figure 2, the swimming duration was significantly higher in the AC-pre-treated groups (60, 12, and 180 mg/kg AC treatment) compared with that of the vehicle-treated group in a dose-dependent manner (15.5, 19.8, and 47.2% increase for the 60, 120, and 180 mg/kg AC-pre-treated groups vs. vehicle-treated group, respectively). These data demonstrate that the AC extract may increase the swimming duration of rats.

### 3.3. AC Extract Preserves Changes in Hemodynamic Parameter and Blood Ions after Exhaustive Swimming 

Exhaustive swimming significantly increased Hct and several blood ions (Mg^2+^, Ca^2+^, Na^+^, and K^+^). However, the changes in the values tended to decrease in rats pre-treated with the AC extract; in particular, 180 mg/kg AC extract pre-treatment significantly inhibited the changes after exhaustive swimming, resulting in similar values to those of the control group (Table 1). Therefore, these results indicate that AC extract pre-treatment was able to maintain blood homeostasis after exhaustive swimming.

### 3.4. AC Extract Attenuates Changes in Energy Metabolism-Related Serum Biomarkers after Exhaustive Swimming

**Figure 3:**
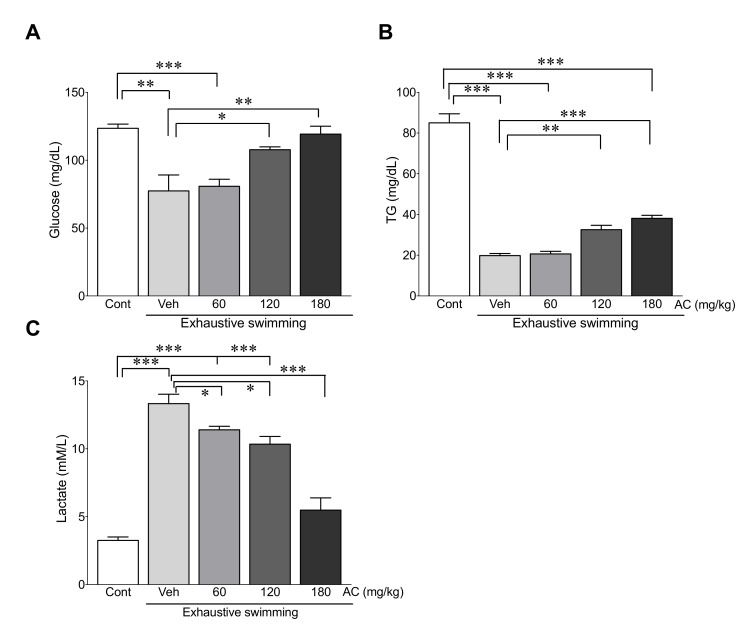
AC extract attenuated changes in energy metabolism-related serum biomarkers from exhaustive swimming. Serum levels of (**A**) glucose, (**B**) TG, and (**C**) lactate were measured in rats after AC pre-treatment for three weeks (*n* = 10 per group). Data are mean ± standard error of mean (SEM). Significance was measured using one-way analysis of variance (ANOVA) followed by Bonferroni’s post hoc test. * *p* < 0.05, ** *p* < 0.01, and *** *p* < 0.001. Cont, control; Veh, vehicle-treated; 60, 120, and 180 AC, 60, 120, 180 mg/kg AC extract-treated groups, respectively. AC, *Aralia continentalis kitagawa* extract; TG, triglyceride.

To evaluate the fatigue effects of the AC extract in rats subjected to exhaustive swimming, fatigue-related biomarkers such as glucose, TG, and lactate were measured. In rats without AC pre-treatment, exhaustive swimming significantly decreased glucose and TG levels compared with those in the control group (from 123.5 to 77.4 mg/dL and 85.0 to 19.8 mg/dL glucose and TG levels, respectively). Conversely, 120 and 180 mg/kg AC pre-treated rats exhibited higher levels of glucose and TG compared with the vehicle-treated group after exhaustive swimming (Figure 3A,B). Lactate levels in the vehicle-treated group after exhaustive swimming were significantly elevated compared with those in the control group (from 3.3 to 13.3 mg/dL). However, 120 and 180 mg/kg AC pre-treatments significantly decreased lactate accumulation compared with levels in the vehicle-treated group (Figure 3C). Therefore, these data suggest that AC attenuated changes in the energy metabolism-related indicators due to exhaustive swimming. 

### 3.5. AC Extract Ameliorates Metabolic Acidosis Due to Exhaustive Swimming 

**Figure 4:**
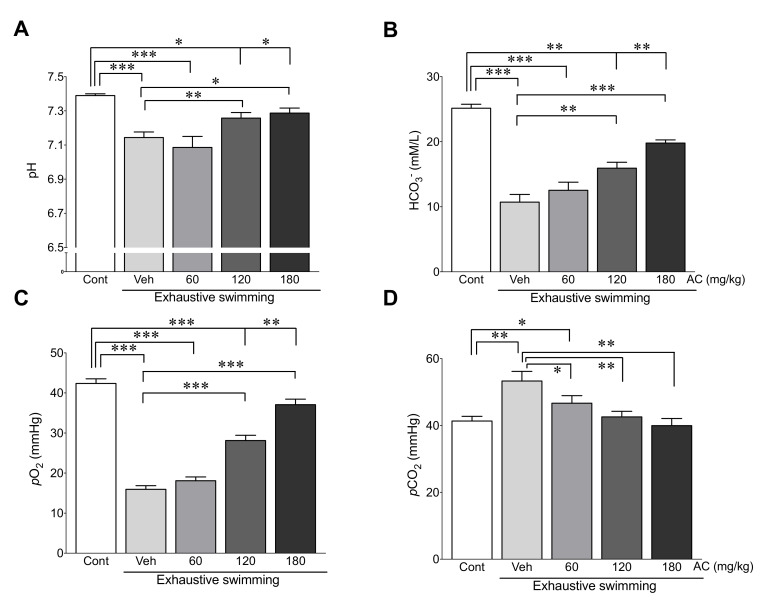
AC extract ameliorates metabolic acidosis due to exhaustive swimming. The blood levels of (**A**) pH, (**B**) HCO_3_^-^, (**C**) *p*O_2_, and (**D**) *p*CO_2_ were determined in rats after AC-pre-treatment for three weeks (*n* = 10 per group). Data are mean ± standard error of mean (SEM). Significance was measured using one-way analysis of variance (ANOVA) followed by Bonferroni’s post hoc test. * *p* < 0.05, ** *p* < 0.01, and *** *p* < 0.001. Cont, control; Veh, vehicle-treated; 60, 120, and 180 AC, 60, 120, 180 mg/kg AC extract-treated groups, respectively. AC, *Aralia continentalis kitagawa* extract; HCO_3_^−^, bicarbonate; *p*O_2_, partial pressure of oxygen; *p*CO_2_, partial pressure of carbon dioxide.

To determine the effects of AC extract on metabolic acidosis after exhaustive swimming, the hemodynamic parameters related to acidosis including blood pH, HCO_3_^−^, *p*O_2_, and *p*CO_2_ were measured in rats with or without AC pre-treatment after exhaustive swimming. The results show that the levels of pH, HCO_3_^−^, and *p*O_2_ were significantly lower in rats without AC pre-treatment after exhaustive swimming compared with those in the control group (3.3, 57.4, and 62.4% decreases in pH, HCO_3_^−^, and *p*O_2_ vs. control group, respectively) (Figure 4A–C). In contrast, levels of *p*CO_2_ significantly increased in rats without AC pre-treatment after exhaustive swimming compared with those in the control group (29.0% increase in *p*CO_2_ vs. the control group) (Figure 4D). Notably, these changes were dramatically attenuated by pre-treatment of AC extracts with 120 and 180 mg/kg (Figure 4). These data indicate that AC prevents metabolic acidosis resulting from exhaustive swimming. 

### 3.6. AC Extract Ameliorates Exhaustive Swimming-Induced Muscle Injury

**Figure 5:**
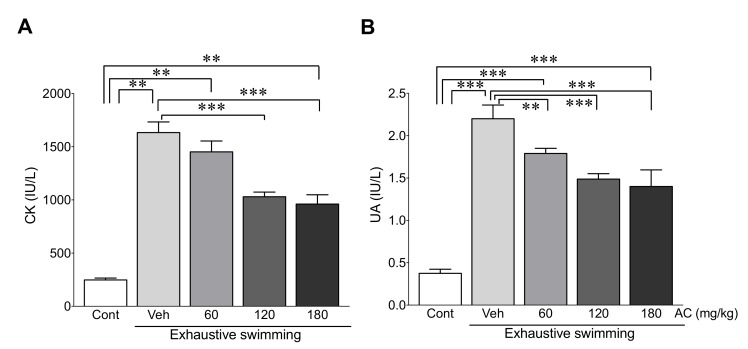
AC extract ameliorated exhaustive swimming-induced muscle injury. The serum levels of (**A**) CK and (**B**) UA were measured in rats after AC pre-treatment for 3 weeks (*n* = 10 per group). Data are mean ± standard error of mean (SEM). Significance was measured using one-way analysis of variance (ANOVA) followed by Bonferroni’s post hoc test. ** *p* < 0.01 and *** *p* < 0.001. Cont, control; Veh, vehicle-treated; 60, 120, and 180 AC, 60, 120, 180 mg/kg AC extract-treated groups, respectively. AC, *Aralia continentalis kitagawa* extract; CK, creatine kinase; UA, uric acid.

To evaluate the effects of AC extract on muscle injury after exhaustive swimming, muscle injury biomarkers such as CK and UA [28] were measured using rats with or without AC pre-treatment after exhaustive swimming. The levels of CK and UA were significantly higher after exhaustive swimming compared with those in the control group (5.6- and 4.9-fold increases in CK and UA vs. the control group, respectively) (Figure 5). However, the increase in the levels of these proteins was significantly ameliorated by AC-pre-treatment with 120 and 180 mg/kg compared with those vehicle-treated rats after exhaustive swimming (62.7 and 58.5% decrease in CK and 67.6 and 63.6% decrease in UA levels in rats pre-treated with 120 and 180 mg/kg AC vs. rats without AC pre-treatment, respectively) (Figure 5). Therefore, these data demonstrate that AC extract mitigates muscle damage after exhaustive swimming. 

### 3.7. AC Extract Suppresses Exhaustive Swimming-Induced Apoptosis

**Figure 6:**
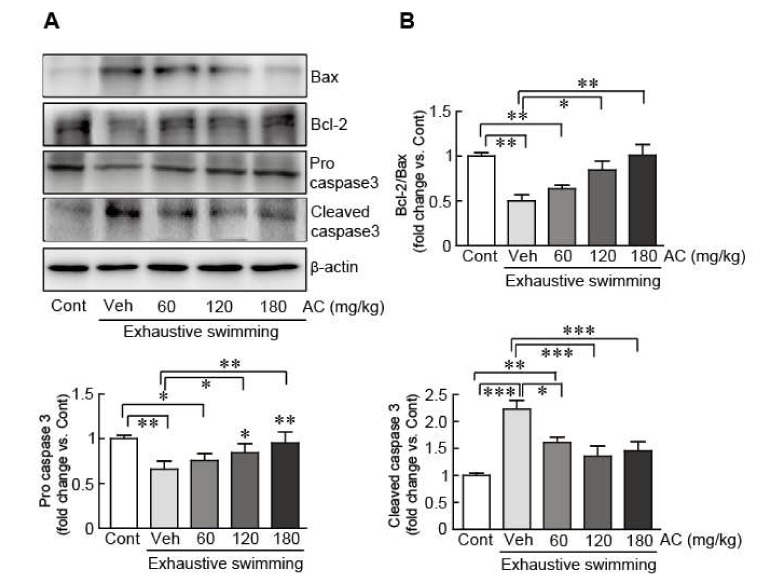
AC extract suppressed exhaustive swimming-induced apoptosis in gastrocnemius muscle. (**A**) Western blot analysis of Bax, Bcl-2, pro-, and cleaved caspase 3 protein expression levels in rats after AC pre-treatment for 3 weeks. (**B**) The protein expression levels were quantified by scanning densitometry. β-actin was used as the loading control. Western blot analysis was performed in triplicate with three independent samples. Data are mean ± standard error of mean (SEM). Significance was measured using one-way analysis of variance (ANOVA) followed by Bonferroni’s post hoc test. * *p* < 0.05 and ** *p* < 0.01, and *** *p* < 0.001. Cont, control; Veh, vehicle-treated; 60, 120, and 180 AC, 60, 120, 180 mg/kg AC extract-treated groups, respectively. AC, *Aralia continentalis kitagawa* extract.

To assess the ameliorative effect of AC extract on exhaustive swimming-induced apoptosis, the expression levels of apoptosis-related proteins including Bax, Bcl-2, pro-, and cleaved-caspase 3, in gastrocnemius muscle were determined. The results show that levels of Bax and cleaved caspase 3 were significantly increased in exhaustive swimming-applied rats without AC-pre-treatment. Pro-caspase 3, an inactive form of caspase 3, and Bcl-2 protein, an anti-apoptotic protein, significantly decreased in exhaustive swimming-applied rats (Figure 6). Notably, the changes in the expression levels of these proteins were significantly attenuated by AC-pre-treatment (Figure 6). Therefore, AC extract can inhibit the apoptosis in gastrocnemius muscle induced by exhaustive swimming. 

### 3.8. AC Extract Attenuates Oxidative Stress Induced by Exhaustive Swimming

**Figure 7:**
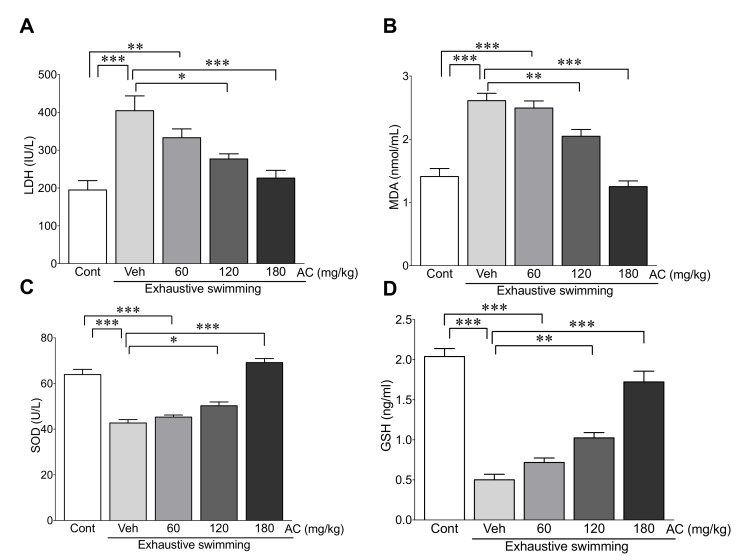
AC extract attenuated oxidative stress induced by exhaustive swimming. The serum levels of (**A**) LDH, (**B**) MDA, (**C**) SOD, and (**D**) GSH were measured in rats after AC pre-treatment for three weeks (*n* = 10 per group). Data are mean ± standard error of mean (SEM). Significance was measured using one-way analysis of variance (ANOVA) followed by Bonferroni’s post hoc test. * *p* < 0.05, ** *p* < 0.01, and *** *p* < 0.001. Cont, control; Veh, vehicle-treated; 60, 120, and 180 AC, 60, 120, 180 mg/kg AC extract-treated groups, respectively. AC, *Aralia continentalis kitagawa* extract; LDH, lactate dehydrogenase; MDA, malondialdehyde; SOD, superoxide dismutase; GSH, glutathione.

To explore whether AC extract has preventive effects against oxidative stress due to exhaustive swimming, the serum levels of oxidative-stress-related biomarkers including LDH, MDA, SOD, and GSH, were measured after exhaustive swimming in rats with or without AC extract pre-treatment. The levels of LDH and MDA were significantly higher in rats without AC-pre-treatment compared with those in the control group (107% and 85% increase in LDH and MDA vs. the control group, respectively). However, the levels of these proteins were significantly preserved in rats pretreated with 120 and 180 mg/kg AC (31.6% and 44.0% decrease in LDH and 21.5% and 52.1% decrease in MDA levels in rats pre-treated with 120 and 180 mg/kg AC vs. rats without AC-pre-treatment, respectively) (Figure 7A,B). For the antioxidants SOD and GSH, the levels were dramatically conserved when rats were pretreated with either 120 and 180 mg/kg AC (17.6% and 61.9% increase in SOD and 104.5% and 243.3% increase in GSH levels in rats pre-treated with 120 and 180 mg/kg AC vs. rats without AC treatment, respectively), although decreased levels were seen in rats without AC-pre-treatment after exhaustive swimming (33.2% and 43.3% decrease in SOD and GSH vs. the control group, respectively) (Figure 7C,D). These data demonstrate that the AC extract possesses antioxidant effects in rats with exhaustive swimming-induced fatigue.

## 4. Discussion

The physiological state of fatigue, which includes idiopathic fatigue, chronic fatigue syndrome, and undefined fatigue, has detrimental effects on health [29]. Fatigue causes muscle pain, impaired memory, disrupted sleep, and other problems [30]. Furthermore, fatigue is associated with a variety of diseases including cancer, hypertension, diabetes, and coronary heart disease [31].

The AC root possesses many pharmaceutical properties including anti-osteoarthritic [32], vasorelaxant [33], anti-inflammatory [17], and anti-cancer [34] activities. In particular, several studies using various disease animal models have demonstrated that AC has antioxidant activities [35]. Indeed, AC prevented the carcinogenesis induced by benzo(α)pyrene through the activation of the antioxidant system in rats [18]. AC also has a protective effect against *tert*-butyl hydroperoxide (*t*-BHP)-induced hepatotoxicity through the inhibition of oxidative stress in both in vitro and in vivo systems [36]. Despite these previous studies of AC’s pharmacological actions, little is known about the anti-fatigue activities of AC in relation to exhaustive exercise. Therefore, the present study evaluated the protective effects of AC roots against the fatigue induced by exhaustive exercise.

Previous studies reported that one of the bioactive compounds in AC extract is chlorogenic acid. Therefore, we assayed the AC extract used in this study and found that it contained 2.24 mg/g of chlorogenic acid. 

To determine the anti-fatigue effects of AC, rats were applied to forced swimming to induce the fatigue that has already been observed in previous studies [7,37]. The results show that pre-treatment with AC effectively increased the forced swimming time. In addition, blood glucose and TG, the primary sources of energy for exercise [38,39], were significantly preserved by AC pre-treatment. Therefore, our results demonstrate that AC could delay the time taken for the depletion of nutrients.

Under normal states, ATP, which is produced by glycolysis through conversion of glycogen into glucose, is utilized as an energy source [40]. However, the supply of energy is changed by conversion of pyruvate to lactate under the anaerobic conditions caused by exhaustive exercise. The accumulation of lactate during exhaustive exercise further causes a reduction in pH and acidosis [6,41], and, consequently, fatigue occurs. Consistent with this, our study showed that the levels of blood glucose, lactate pH, HCO_3_^−^, and *p*O_2_ decreased and *p*CO_2_ increased due to exhaustive swimming. Importantly, these changes were dramatically attenuated by AC-pre-treatment. These findings demonstrate that AC has a preventive role against the metabolic acidosis resulting from exhaustive exercise.

The present study showed that several blood ion levels were affected by exhaustive swimming. Indeed, the relationship between blood ions and exercise has been extensively studied [42]. Mg^2+^ and K^+^ ions are closely associated with numerous muscle functions encompassing contractility, energy production, oxidative stress, and electrolyte balance [7]. In particular, Mg^2+^ ions are re-distributed to adjust metabolism for the maintenance of muscle contractility during exercise [43,44,45]. In this regard, our previous study found that blood Mg^2+^ ions increased after exhaustive swimming. Mg^2+^ ions also have a positive relationship with lactate, UA, LDH, and CK, but a negative relationship with glucose and TG [7]. Other ions such as K^+^, Ca^2+^, and Na^+^ were also shown to increase during exercise [7,46]. The present study demonstrated that AC-pre-treatment attenuated the increase in these ions due to exhaustive swimming.

The reduction of blood flow due to exhaustive exercise leads to an increased Hct value, which further causes impairment to the oxygen supply and energy production [47]. Therefore, Hct has been used as an indicator of the degree of fatigue caused by exercise. Our results show that AC-pre-treatment preserved Hct levels during exhaustive swimming.

Additionally, exhaustive exercise is closely associated with muscle and renal damage, which can affect physical performance [48]. Serum CK and UA are considered crucial biomarkers of various types of muscle damage such as cardiac diseases, muscular dystrophy, and acute renal failure [28]. Therefore, these enzymes have been widely used as indicators of muscle and renal damage caused by exhaustive exercise [43]. Regarding this, we measured the serum levels of CK and UA to assess whether AC can attenuate muscle damage during exhaustive exercise, and, as expected, treatment with the extract effectively reduced serum CK and UA levels after exhaustive exercise. Therefore, AC may prevent muscle and renal damage due to the exhaustive exercise.

Apoptosis is defined as a programmed cell death that ensures cellular homeostasis; it is sensitive to the intracellular redox environment and, thus, is implicated in oxidative stress [49]. Exhaustive exercise causes augmented apoptosis, which induces DNA fragmentation and altered apoptosis-related gene and protein expression including that of Bax, Bcl-2, and the caspases [50,51]. Therefore, apoptosis has a detrimental effect on nuclear and mitochondrial integrity and, consequently, triggers skeletal muscle damage that causes fatigue under exhaustive exercise. Here, we demonstrated that AC extract effectively attenuated the increases in apoptotic-inducible proteins including Bax and pro-caspase 3. However, the treatment decreased the levels of cleaved-caspase 3, an apoptotic-inducible protein, and Bcl-2, an anti-apoptotic protein, in gastrocnemius muscles induced by exhaustive swimming, demonstrating that AC can attenuate fatigue by inhibiting apoptosis in gastrocnemius muscles.

We sought to elucidate the protective effects of AC against the oxidative stress induced by exhaustive exercise and the underlying mechanisms involved. Intense physical exercise causes an imbalance between oxidants and antioxidant systems because of the release of oxygen-derived free radicals such as ROS, further contributing to the induction of muscle fatigue [37]. Indeed, a wealth of evidence has shown that oxidative stress is closely associated with the fatigue of exhaustive exercise [52]. During exhaustive exercise, oxygen demand is increased and blood flow in skeletal muscle changes. These changes cause free radical production and disturbances in muscle homeostasis, leading to oxidative damage in skeletal muscle, a subsequent inflammatory response, and the production of cytokines, further contributing to muscle fatigue. Previous studies using animal models also revealed that high-intensity exhaustive exercise resulted in a reduction in the levels of the antioxidants SOD and GSH and elevation in the levels of MDA, a lipid peroxidation by-product, as a result of oxidative damage [53,54]. Thus, the preservation of antioxidant systems against oxidative stress could be an effective method of improving exercise ability during exhaustive exercise. The present study revealed that the increase in the levels of the pro-oxidants LDA and MDA and the decrease in the levels of the antioxidants SOD and GSH due to exhaustive exercise were effectively reversed by pre-treatment with AC. These results indicate that the anti-fatigue effect of AC is mediated by the modulation of oxidative stress following exhaustive exercise.

## 5. Conclusions

In conclusion, the findings of the current study suggest that AC attenuates the physical fatigue induced by exhaustive exercise by ameliorating metabolic acidosis and possesses antioxidant protection capacity. Therefore, we propose AC as a potential substance for the prevention and treatment of physical fatigue from exhaustive exercise. 

## Figures and Tables

**Table 1:** *Aralia continentalis kitagawa* (AC) extract preserved changes in hemodynamic parameter and blood ions after exhaustive swimming.

Parameters		Exhaustive Swimming			
	Cont	Veh	60	120	180 AC (mg/kg)
Hct (%)	36.9 ± 0.6	43.1 ± 0.8 ^###^	41.9 ± 0.6 ^###^	39.6 ± 0.5 ^#,^**	37.2 ± 0.5 **
Mg^2+^ (mmol/L)	0.52 ± 0.08	0.62 ± 0.01 ^###^	0.57 ± 0.01 ^#^	0.56 ± 0.02 *	0.54 ± 0.01 **
Ca^2+^ (mmol/L)	1.25 ± 0.03	1.40 ± 0.01 ^##^	1.34 ± 0.01 ^#^	1.31 ± 0.03 ^#^	1.29 ± 0.02 **
Na^+^ (mmol/L)	139.9 ± 1.0	146.1 ± 0.9 ^##^	145.3 ± 1.0 ^##^	141 ± 2.2	140.4 ± 0.7 *
K^+^ (mmol/L)	4.5 ± 0.3	5.2 ± 0.2 ^##^	4.9 ± 0.1 ^#^	4.6 ± 0.4	4.5 ± 0.1 *
BW (g)	362.9 ± 7.4	345.6 ± 2.0	330.2 ± 3.4 ^###^	329.3 ± 1.9 ^###^	324.2 ± 3.2 **

Data are mean ± standard error of mean (SEM). Significance was measured using one-way analysis of variance (ANOVA) followed by Bonferroni’s post hoc test. ^#^*p* < 0.05, ^##^ *p* < 0.01, and ^###^ *p* < 0.001 vs. control group. * *p* < 0.05 and ** *p* < 0.01, vs. vehicle-treated group. Cont, control; Veh, vehicle-treated; 60, 120, and 180 AC, 60, 120, 180 mg/kg AC extract-treated groups, respectively. AC, *Aralia continentalis kitagawa* extract; Hct, hematocrit. BW, body weight.
